# GENDER DIFFERENCE IN USING DIGITAL HEALTH TECHNOLOGIES AMONG OLDER ADULTS WITH CANCER: A PILOT QUALITATIVE STUDY

**DOI:** 10.1093/geroni/igad104.2683

**Published:** 2023-12-21

**Authors:** Misun Hwang, Youmin Cho, Katie Gahn, Milisa Manojlovich, Yun Jiang

**Affiliations:** University of Michigan School of Nursing, Ann Arbor, Michigan, United States; The University of Texas Health Science Center at Houston, Houtston, Texas, United States; University of Michigan, Ann Arbor, Michigan, United States; University of Michigan, Ann Arbor, Michigan, United States; University of Michigan, Ann Arbor, Michigan, United States

## Abstract

Digital technologies hold promises to improve health and care delivery for various populations, including older adults with cancer. However, older adults’ adoption of digital health technology is less optimal. While age and gender can interfere with people’s technology behavioral intention, it is unknown how gender differences may influence older adults with cancer’s technology use experiences. This pilot qualitative study investigated gender differences in older adults’ experiences using digital technologies to manage their cancer and care. Semi-structured interviews were conducted with 16 older adults with breast, colorectal, lung, or prostate cancer (men=7 and women=9) aged 65 to 78 years. Thematic data analysis revealed that older men favored the adjustability and efficiency of using technology to save time and cost compared to in-person visits. Also, they perceived that technology facilitated their adherence to treatment plans. Older women, however, preferred technology-supported individualized care and perceived being able to review their medical notes as beneficial. Women were comfortable using technology to timely communicate with their trusted care providers. However, they preferred to see their providers in person if they experienced severe condition changes or unanticipated symptoms. Despite all these different preferences, both genders were comfortable using video calls with their providers, using the portal to keep track of their medical records and share decision-making with clinicians. These pilot results demonstrated gender differences in experiences and perceptions of using digital technology among older adults with cancer and suggested integrating gender preferences into technology design and development to facilitate personalized user experience and improve patient engagement.

